# State-of-the-Art and Future Directions in Structural Proteomics

**DOI:** 10.1016/j.mcpro.2025.101065

**Published:** 2025-09-03

**Authors:** Lotta J. Happonen, Markku Varjosalo

**Affiliations:** 1 Division of Infection Medicine, Department of Clinical Sciences Lund, Faculty of Medicine and Science for Life Laboratory, Lund University, Lund, Sweden; 2 Institute of Biotechnology, HiLIFE, Faculty of Medicine, University of Helsinki, Helsinki, Finland

**Keywords:** systems structural proteomics, XL-MS, HDX-MS, LiP-MS, integrated structural biology

## Abstract

Structural proteomics has undergone a profound transformation, driven by the convergence of advanced experimental methodologies and computational innovations. Cutting-edge mass spectrometry (MS)-based approaches, including cross-linking MS (XL-MS), hydrogen-deuterium exchange MS (HDX-MS), and limited proteolysis MS (LiP-MS), now enable unprecedented insights into protein topology, conformational dynamics, and protein–protein interactions. These methods, complemented by affinity purification (AP), co-immunoprecipitation (co-IP), proximity labeling (PL), and spatial proteomics techniques, have expanded our ability to characterize the structural proteome at a systems-wide scale. Integration with electron cryo-microscopy (cryo-EM), cryo-electron tomography (cryo-ET), nuclear magnetic resonance (NMR) spectroscopy, X-ray crystallography, and small-angle X-ray/neutron scattering (SAXS/SANS) methods has further driven the field of integrative structural biology. These methods, in conjunction with AI-driven predictive models such as AlphaFold and RoseTTAFold, enable the high-resolution modeling of protein complexes and dynamic assemblies, bridging the gap between static structures and real-time conformational changes. This review explores the current state-of-the-art in structural proteomics, with a focus on methodological advances and the integration of XL-MS, HDX-MS, and LiP-MS with methods in structural biology. We further discuss the application of structural proteomics in deciphering disease mechanisms, identifying therapeutic targets, and guiding drug discovery, with these techniques poised to revolutionize precision medicine. Future directions emphasize fully integrative, multimodal approaches that unify experimental and computational paradigms, fostering a holistic understanding of the human proteome.

## Highlights

• XL-MS, HDX-MS, and LiP-MS reveal protein structure, dynamics, and interaction networks.
• Integration with cryo-EM/ET, NMR, crystallography, SAXS/SANS, and AI refines models.
• Structural proteomics links molecular architecture to disease mechanisms and therapies.
• Systems structural proteomics connects molecular detail to cellular and organismal function.

## Graphical Abstract

**Figure:**
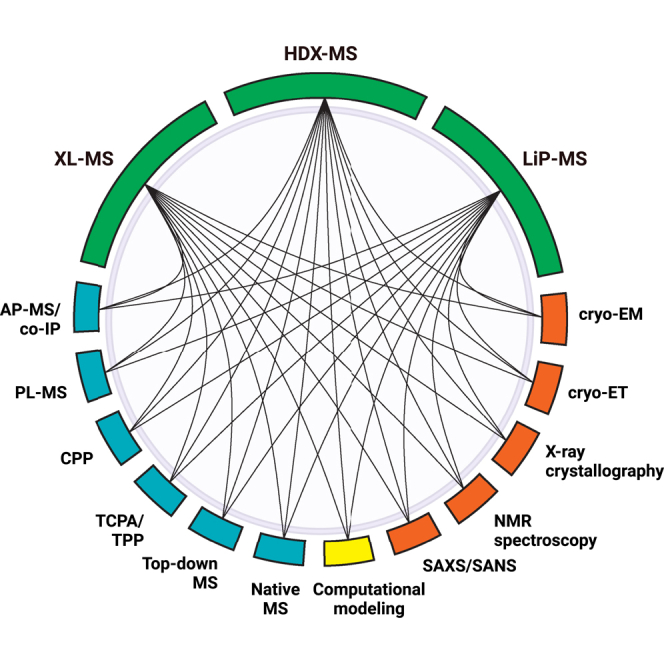

## In Brief

In this review we survey state-of-the-art structural proteomics, focusing on XL-MS, HDX-MS and LiP-MS and how they integrate with cryo-EM/ET, NMR, SAXS/SANS, top-down and native-MS, and AI-based modeling to define protein structures, interactions and dynamics across scales. We outline systems-level applications in health and disease, from mechanism to target discovery, and propose future multimodal workflows that combine spatial proteomics and *in situ* analyses to enable proteome-wide, context-aware models for precision medicine.

**Fig. 1:**
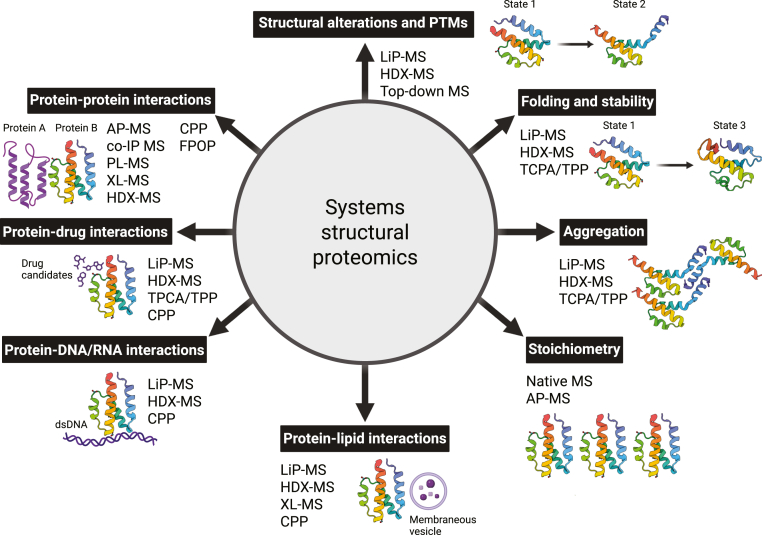
**Overview of structural proteomics methods and their most common applications.** Multiple structural proteomics methods are applied across the molecular scale of life for the studies of key cellular events including but not limited to protein–protein, protein–drug, protein–nucleic acid and protein–lipid interactions, protein folding and stability, structural protein alterations, the analysis of post-translational modifications (PTMs) and protein aggregation as well as to address questions relating to protein stoichiometry in larger complexes. These studies span cellular biology across the scale of smaller protein complexes in solution (mainly HDX-MS) to larger protein machineries *in vivo* (XL-MS, LiP-MS) and in combination with other methods in proteomics (AP-MS, co-IP, PL-MS, TPCA/TPP, top-down MS and native MS). Protein A: generic protein Z (Biorender icon), protein B: complement system C5a-desArg; state 1 - PDB ID: 4P3B (323), state 2 - PDB ID: 3HQA (324), and state 3 - PDB ID: 1KJS (325). Created in https://BioRender.com.

The structural characterization of proteins and the complexes they form is paramount to understanding the molecular basis of biological processes and disease mechanisms. The field of integrated structural biology has steadily advanced, enabled by a growing repertoire of techniques that are now collectively used to decipher the molecular mechanisms of events central to all life. State-of-the-art MS-based structural proteomics approaches, including cross-linking MS (XL-MS) (1, 2, 3), hydrogen-deuterium exchange MS (HDX-MS) (4, 5) and limited proteolysis MS (LiP-MS) (6, 7), offer insights into protein structure, dynamic conformational changes, and protein–protein interactions (PPIs). These strategies, together with other proteomics methods used for mapping PPIs (affinity purification (AP), co-immunoprecipitation (co-IP), proximity labeling (PL), thermal proteome profiling (TPP), chemical footprinting, fast photochemical oxidation of proteins (FPOP), and covalent protein painting (CPP) (8, 9) are currently being applied across the molecular scale of smaller protein complexes *in vitro* to larger protein machineries, organelles, and entire cellular proteomes *in vivo* (10) (Fig. 1). Moreover, the emerging field of systems structural proteomics integrates these large-scale analyses with computational modeling to uncover proteome-wide interplay of protein complexes under physiological and pathological conditions. In parallel, techniques such as cryogenic electron microscopy (cryo-EM) and electron cryo-tomography (cryo-ET) (11, 12, 13) reveal the structures and compositions of large, heterogeneous complexes, and membrane-associated assemblies, while yet other techniques such as nuclear magnetic resonance (NMR) spectroscopy, X-ray crystallography, and small-angle X-ray and neutron scattering (SAXS and SANS, respectively) continue to refine the toolkit of methods in integrated structural biology. On the computational front, the advent of artificial intelligence (AI)-based model prediction (*e.g.*, AlphaFold, RoseTTAFold) (14, 15, 16, 17) has revolutionized the generation of high-accuracy protein models, enabling the integration of these predicted models with experimental data to resolve complex assemblies and dynamic landscapes. In this review, we summarize the current state-of-the-art in structural proteomics and discuss the methodological and technical approaches for successful XL-MS, HDX-MS, and LiP-MS experiments. We showcase how complementary techniques can be used together with XL-MS, HDX-MS, and LiP-MS, and what the synergies of combining these methods are. We further emphasize the need for integrative and multimodal approaches that bridge the experimental and computational fields, ultimately advancing our ability to decode the structural proteome at a systems-wide level and to advance our understanding of the human proteome in health and disease.

## Experimental Approaches in Peptide-Level Structural Proteomics Methods

A central goal of structural proteomics is to characterize protein structures, dynamics, and interactions at scale and *in vivo*-like conditions. Mass spectrometry (MS)-based methods have emerged as particularly powerful for probing structures of large, heterogeneous, or difficult-to-purify complexes. Traditional and widely used methods in structural proteomics encompassing XL-MS, HDX-MS and LiP-MS all rely on bottom-up, peptide-based mass spectrometry (18). In addition to these core MS techniques, several specialized methods have been developed to probe protein–protein interactions and conformational dynamics.

### Basics of Bottom-Up Proteomics

Bottom-up proteomics is the most widely used MS-based strategy for large-scale protein identification and quantification. In a standard bottom-up proteomics experiment, proteins in a sample (*e.g.*, cell lysate, tissue extract, liquid biopsy, protein complex) are proteolytically digested to peptides (18). These peptides are separated by liquid chromatography (LC) and analyzed by tandem mass spectrometry (MS^2^or MS^3^). The resulting peptide-spectrum matches (PSMs) are mapped back to their parent protein(s) via database matching (in case of DDA or library-free DIA, data-dependent acquisition or data-independent acquisition, respectively) and/or spectral library matching (for library-based DIA) (10, 18, 19, 20). A key limitation of bottom-up proteomics is the peptide-to-protein inference problem: multiple proteins may share peptides, complicating unambiguous protein identification and variant or isoform detection.

Despite its inherent peptide-level granularity, bottom-up proteomics is still the most widely adopted discovery platform as it tolerates samples of extreme complexity and dynamic range (cell lysates, tissues, liquid biopsies) and requires only micrograms of unfractionated input. High-resolution instruments and advanced data processing software (*e.g.*, MaxQuant (21), Proteome Discoverer, FragPipe (22), Spectronaut (23), and DIANN (24)) enable accurate mass measurement of thousands to tens of thousands of peptides in a single LC-MS/MS run. DIA-MS can routinely identify and quantify >10,000 proteins in a single analysis (25, 26), often providing comprehensive proteome coverage. Quantitative DDA and DIA methods include stable isotope labeling (SILAC, TMT/iTRAQ) (27, 28, 29) or label-free quantitation (LFQ), facilitating relative or absolute protein abundance measurements.

In contrast, top-down or intact/native MS workflows struggle when the analyte increases to hundreds of coexisting proteoforms present in the same spectrum. Large proteins and multi-subunit assemblies ionize less efficiently, produce congested charge-state envelopes, and often cannot be fully desolvated, leading to peak broadening and mass-assignment ambiguities. Specialized instrumentation (ultra-high-m/z Orbitraps or FT-ICRs, modified ion optics) and mg amounts of highly purified material are commonly required, and even then, data analysis is hampered by overlapping isotopic distributions, incomplete fragment coverage, and the lack of high-throughput bioinformatic pipelines. Consequently, for global proteome surveys, especially for complex samples, bottom-up LC-MS/MS is more sensitive, has a higher throughput and analytical robustness, while top-down and native approaches are most powerful when focused on well-defined targets or isolated complexes (30).

Bottom-up proteomics is integral to structural proteomics by allowing for the: (1) mapping of cross-linked peptides, *i.e.* in identifying cross-linked peptides that report distance constraints; (2) HDX-MS and LiP-MS analyses, of which both rely on bottom-up workflows to pinpoint deuterium incorporation or protease cleavage sites at peptide-level resolution; and (3) in the analysis of post-translational modification (PTMs), where bottom-up proteomics excels at locating PTMs (*e.g.*, phosphorylation, glycosylation, and ubiquitination) on peptides and analyzing sample micro-heterogeneity. Bottom-up proteomics remains the foundation of proteome-scale analyses, both in quantitative proteomics studies but also in structural proteomics projects, with future developments focused on deeper coverage, higher dynamic range, and refined structural insights.

### Cross-Linking Mass Spectrometry (XL-MS)

**Fig. 2:**
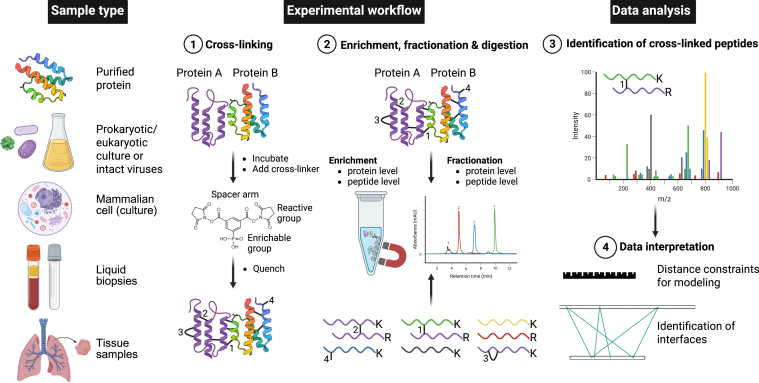
**Overview of the sample requirements, experimental workflow and data analysis for cross-linking mass spectrometry.** XL-MS can be applied for a broad variety of sample types, including purified proteins or protein complexes in solution, prokaryotic and lower eukaryotic cultures, intact viruses, mammalian cells (in culture), diverse liquid biopsies and isolated tissue samples. In the experimental setup, the sample to be cross-linked is incubated in a suitable buffer at a suitable temperature, the cross-linker is added and activated by *e.g.* ultraviolet (UV)-light if applicable, followed by quenching of the reaction. In a typical reaction, the generated cross-links are (1) inter-protein cross-links, (2) intra-protein cross-links, (3) loop-links on one peptide, and (4) dead-end cross-links (*lower panel*). For modeling and data integration with other methods, the interprotein and intraprotein cross-links are the most useful ones. After cross-linking, the sample is proteolytically digested to peptides, with an optional enrichment or fractionation step at either the protein or peptide level. The cross-linked peptides are identified and quantified by liquid-chromatography tandem mass spectrometry (LC-MS/MS; at the MS^2^ or MS^3^ level, the latter in case of MS-cleavable cross-linkers), followed by data analysis using dedicated software and data interpretation. The typical data generated in an XL-MS experiment yields distance constraints defined by the spacer arm length of the cross-linker used and identifies cross-linked residues between two peptides, useful for modeling. The cross-linking reagent depicted is PhoX (35). Protein A: generic protein Z (Biorender icon), protein B: complement system C5a-desArg; PDB ID: 4P3B (323). Created in https://BioRender.com.

Cross-linking mass spectrometry is a powerful method to probe interacting peptides or peptides in close proximity to each other in three-dimensional (3D) space. Such information of the spatial distance constraints between a given set of peptides is invaluable for the modeling of proteins, and the AI-based modeling and docking of protein complexes (31). XL-MS can be applied across the entire molecular scale from individual proteins in solution to larger protein machineries and entire organelles and intact cells (Fig. 2 and Textbox 1), as covered in depth in several relatively recent reviews (32, 33, 34). In a standard XL-MS experiment, the sample of interest is incubated together with a hetero- or homobifunctional chemical cross-linker to covalently link specific amino acid side chains in close spatial proximity. The spatial proximity is determined by the spacer-arm of the cross-linker (Fig. 2). Once cross-linked, the complexes are digested into peptides and analyzed by bottom-up MS (18), allowing for the identification of the cross-linked peptides and residues that report on intra- and intermolecular interactions (Fig. 2).

Technological advances have expanded the repertoire of cross-linkers, and the previous pillars of XL-MS-based experiments, BS3 and DSS, are to an increasing degree being replaced by enrichable cross-linkers. Such cross-linkers utilize alkyne-tags and click chemistry to biotin-groups or tags which can be enriched for by immobilized metal affinity chromatography (IMAC) or other solid matrix. These cross-linkers include PhoX (35) (Fig. 2), tBu-PhoX (36), and *e.g.*, DSBSO-derivatives (37, 38), which allow for an improved sensitivity and increased coverage in case of proteome-wide cross-linking. Additional complexity reduction and enrichment of cross-linked peptide species, especially in large-scale, organelle, or cellular level cross-linking studies, can be achieved by combining the use of enrichable cross-linkers with size exclusion chromatography (SEC), strong cation exchange (SCX), or high-pH reverse phase fractionation. In addition to the use of enrichable cross-linkers with specific reactive groups targetable with beads, other enrichment methods, such as AP or co-IP before proteolytic digestion, increase the sensitivity by enriching for the complex(es) of interest (Fig. 2).

MS-cleavable reagents, on the other hand (39), including DSBSO-derivatives (37, 38), DSSO (40), and DSBU (41), all improve specificity and confidence in cross-link assignments, mainly when investigating complex biological systems. The main benefits of MS-cleavable reagents are the improved fragmentation, yielding a more complete and unambiguous identification of cross-linked peptides, and the easier analysis of their masses and sequences.

In addition to increasing the repertoire of cross-linkers to include enrichable and MS-cleavable ones, the standard homobifunctional lysine (K)-specific reaction chemistry is complemented with other chemistries, such as acidic-residue (aspartate (D) and glutamate (E)) specific cross-linkers, including ADH (42), PDH (42), and Diazoker 1 (43); sulfhydryl-reactive chemicals such as SMCC and derivatives, as well as SPDP and derivatives. The DBB and DBMT reagents cross-link tyrosines and the latter histidines under specific conditions (44, 45). Yet others include photoreaction-activated cross-linkers, including SDA and derivatives, with even broader reaction chemistries. Such cross-linkers with alternative reaction chemistries to lysine-specific ones increase the mappable protein-protein interaction space in complex samples and provide complementarity to mapping interacting interfaces even in low complexity samples where lysines are scarce.

Automated data analysis pipelines, including software such as pLink3 (46), xiSearch (47), and MaxLynx (48) and improved informatics platforms, now assist in handling the complexity of XL-MS datasets, ensuring robust and reproducible structural insights. Several visualization tools have been developed for the analysis and visualization of XL-MS data, including xiNET (49), xiSPEC (50) and xiVIEW (51), as well as plugins developed for ChimeraX (52) & pyMOL (The PyMOL Molecular Graphics System, Version 3.0 Schrödinger, LLC) (53). Integration of XL-MS workflows with modeling and docking algorithms (*e.g.* AlphaLink, Sidewinder) further extends the repertoire of available software and applications in life science (54, 55, 56).

### Current State-of-the-Art in XL-MS

The field of XL-MS and its applications in biological research is (close to) reaching its maturation, and the main challenges ahead lie in applying this method to more complex systems and challenging research problems. On the data analysis and interpretation side, the XL-MS community still needs to improve data reliability and decrease false discoveries, especially for novel PPIs in complex backgrounds. There is, however, progress in this direction as highlighted by a recent summary of the increased reliability, transparency, and accessibility in XL-MS working toward a common set of field recommendations and standardization (34) as well as the first community-wide, comparative XL-MS study (57). Ground-breaking research applying XL-MS during the past decade include *e.g.* research on ribosomes and the translation of proteins (58), describing of an assembly-coupled conformational switching the proteasome (59), advances on describing the structural organization of the mitochondrial proteome (60, 61, 62), structural studies on the nuclear pore complex (63, 64, 65), deciphering the interactome of motile cilia (66) as well as *in vivo* and *in situ* cross-linking of the entire cellular proteome (54, 55, 67, 68, 69).

### Future Developments in XL-MS

The most interesting challenges for XL-MS in the next 5 years lie in providing increased examples using *in vivo* XL-MS to study key cellular events and pathways (3, 60, 69, 70, 71, 72) and combining *in vivo* XL-MS with cryoET, spatial proteomics, other proteomics (73, 74) and imaging methods as well as in-cell NMR (75, 76, 77). Whereas examples in this area already exists, we envision that the combination of these methods together with ongoing efforts to catalogue the organization of the entire (human cellular) proteome, such as π-HuB (proteomic navigator of the human body) (78), will allow us to gain unprecedented, detailed insight into PPIs and PPI-related disease mechanisms. Moreover, challenges for XL-MS include increasing the integration of XL-MS with ML- and AI-based modeling and docking; both on the level of data integration (79, 80, 81, 82, 83, 84, 85, 86) but also on the level of developing novel software (15, 16, 54, 87), including software for modeling dynamic regions and complexes (88). Further challenges in developing computational tools include developing new software that combine raw data analysis with modeling and docking “on the go” (56, 89). Additional possibilities lie in the increased application of molecular dynamics (MD) simulations based on complexes and interfaces identified from XL-MS data, as demonstrated by recent examples (80, 81, 90, 91, 92), sometimes even on the level of describing the organellar or cellular proteome (82, 83). Experimental and technological developments include migrating from DDA-based XL-MS to DIA-based XL-MS (31, 93, 94). Quantitative (4D-)DIA-based XL-MS offers increased throughput with higher data coverage, increased reproducibility, and accurate quantification for deeper proteome coverage in complex samples as compared to DDA-based XL-MS workflows (93, 94). While quantitative XL-MS is not yet as mature as PPI mapping, the first structural applications of quantitative XL-MS are currently being described (95, 96, 97). Finally, we expect to see an increasing number of examples of protein–nucleic acid, protein–lipid/membrane, and protein–glycan (98) XL-MS studies.

### Hydrogen-Deuterium Exchange Mass Spectrometry (HDX-MS)

**Fig. 3:**
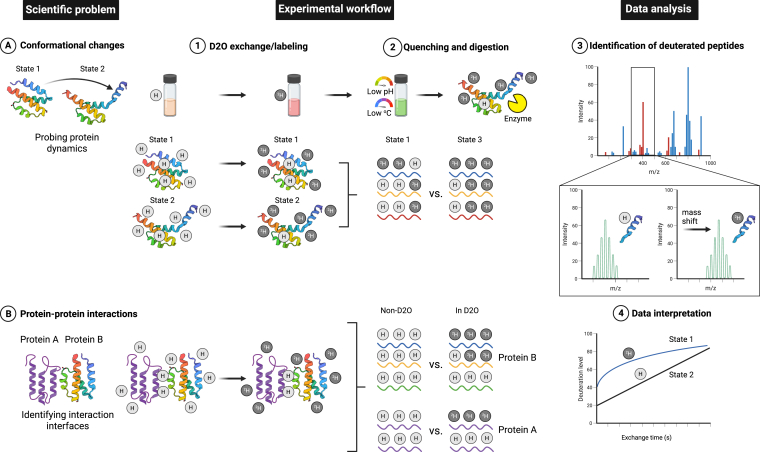
**Overview of the scientific problems addressed, experimental workflow, and data analysis for hydrogen-deuterium exchange mass spectrometry.** HDX-MS can be applied on the level of one or a few purified proteins in solution, and is mainly used to address protein stability, folding and dynamics (*A*, conformational changes) or to identify interaction interfaces or allosteric changes occurring during protein interaction (*B*, protein-protein interactions). For both scientific problems addressed, in the experimental setup, the sample(s) are first incubated in a buffer containing hydrogen, and subsequently in a buffer containing deuterium for D_2_O exchange/labeling for a defined set of time points, during which backbone hydrogens exchange with deuterium. The reactions are quenched at low pH and low temperature to prevent back-exchange, and proteolytically digested to peptides using an enzyme with a low pH optimum. The peptides are identified and quantified by liquid-chromatography tandem mass spectrometry (LC-MS/MS; MS^2^), followed by data analysis using dedicated software and data interpretation. The typical data generated in an HDX-MS experiment yields peptide level information on deuterium uptake over time. Protein A: generic protein Z (Biorender icon), protein B: complement system C5a-desArg; state 1 - PDB ID: 4P3B (323), state 2 - PDB ID: 1KJS (325). Created in https://BioRender.com.

In contrast to XL-MS, which can be applied across the entire molecular scale of simple samples in solution to intact cells, hydrogen-deuterium exchange mass spectrometry can mainly be applied on the level of one to a few individual proteins; however, recent publications highlight HDX-MS applications on complex samples, such as cell lysates and protein libraries (99, 100) (Fig. 3). HDX-MS is a powerful method to probe for disordered or unfolded regions in a protein (101), such as histone tails (102, 103, 104), to probe for stability and conformational changes upon, *i.e.*, small-molecule or nucleic acid ligand-binding, and to determine the interaction interfaces between two or a few proteins (5, 105); often at a higher residue-level resolution than XL-MS (106). Notably, the increased residue-level resolution in HDX-MS experiments as compared to XL-MS (or LiP-MS, see below) stems from the fact that, whereas XL-MS and LiP-MS are bottom-up methods performed at the peptide level, deuterium incorporation at amide bonds at the single-residue level in HDX-MS experiments can be achieved by soft fragmentation methods (electron transfer and capture dissociation, ETD and ECD) (107, 108, 109).

With recent advances in automation (110, 111), software, new applications such as subzero chromatography (112), lipid filtration especially for membrane proteins (113) as well as field-specific guidelines (4, 5, 114), this methodology continues to expand in both academic and industrial settings, providing deeper insights into protein function, stability, and interactions. HDX-MS measures the rate at which backbone amide hydrogens exchange with deuterium in solution (Fig. 3). Exchange occurs faster in flexible, solvent-exposed regions and more slowly in buried and folded regions or domains, allowing HDX-MS to pinpoint structural changes at near-residue-level resolution. HDX-MS can provide a more dynamic view of a protein or protein complex in solution, as opposed to the information acquired by XL-MS, especially in relation to small conformational shifts in domains, relocation or increased protections of disordered regions or the binding of a ligand. In a standard HDX-MS experiment, the protein(s) or protein-ligand complexes initially present in a buffer containing hydrogens, are diluted into a deuterated buffer for varying time periods (*e.g.* 30s, 300s, and 3000s), then rapidly acidified and cooled to quench the exchange reaction. Faster deuterium uptake and hence reaction times or dynamics can be captured when using rapid-mixing devices for millisecond labeling (114). The protein(s) are digested into peptides (commonly with an acid protease such as pepsin) and analyzed by bottom-up MS. The mass shift of each peptide reveals the extent of deuterium uptake per given peptide, indicating localized conformational flexibility (increased deuterium uptake with increased incubation time in deuterated reaction buffer) or protection (no change in deuterium-uptake pattern upon increased incubation) (Fig. 3). Comparative HDX-MS experiments between an apo (ligand-free) protein state and a ligand-bound state or a PPI are used to identify structural changes and interaction dynamics (115, 116). Changes in peptide-level deuterium uptake patterns in these two states reveal insights into conformational changes and dynamics associated with ligand-binding or a PPI. Careful control of pH, temperature, and back-exchange is essential and replicate time points establish reproducibility; hence a robotic sample preparation station is frequently used for increased throughput (99). The method’s utility spans from mapping antibody epitopes (117, 118, 119), identifying allosteric pathways (120, 121), characterizing dynamic complexes (122, 123, 124), and following protein-folding transitions (125, 126). Automated data analysis pipelines including software such as HDExaminer (https://massspec.com/hdexaminer/), HDX-Analyzer (127), Mass Spec Studio (128) visualization tools such as HdgraphiX (129) and HDX-Viewer (130), and improved informatics platforms; the majority of the above as recently reviewed by Stofella *et al.* (131), now assist in handling the complexity of HDX-MS datasets, ensuring robust and reproducible insight into protein dynamics, conformational alterations and interaction interfaces. Integration of HDX-MS workflows with modeling and docking algorithms (*e.g.* RosettaHDX, AI-HDX) further extends the repertoire of available software and applications in life science (132, 133, 134).

### Current State-of-the-Art in HDX-MS

As for XL-MS, HDX-MS and the methodology are reaching its maturation, and the main challenges ahead evolves around applying this method on more complex systems and challenging research problems. For HDX-MS, there exists a common set of field recommendations and standardization (5). Ground-breaking research applying HDX-MS during the past decade include *e.g.* insights into protein folding and misfolding, where HDX-MS has been instrumental in elucidating protein folding pathways and identifying transient intermediates in neurodegenerative diseases such as Alzheimer’s and Parkinsons disease (135, 136, 137). In addition to determining protein folding and misfolding, HDX-MS has been central in enhancing our understanding of PPIs by mapping interaction interfaces and detecting conformational changes upon complex formation. The field of drug discovery and the pharmaceutical industry has leveraged HDX-MS to map protein–ligand interactions with high precision, advancing drug discovery, and aiding the development of drugs with improved efficacy and specificity, such as targets for diseases in lipid metabolism (138), cancers (139), and G protein-coupled receptors (GPCRs), which are key drug targets due to their involvement in many physiological processes, as recently reviewed (140). State-of-the-art applications, moreover, encompass the mapping of antibody-epitopes by HDX-MS (106, 117), allowing for the development of novel antibody-based drugs, as described, for example, for SARS-CoV-2 (141) and antibody-drug conjugates against certain tumors (142).

### Future Developments in HDX-MS

The challenges of HDX-MS in the next 5 years lie in providing increased examples with faster reaction times (from seconds to subsecond and milliseconds) (114, 135, 143), possibly in connection with developing improved mixing devices to further decrease the reaction times, and allowing for the study of fast dynamic reactions, such as the folding of proteins in general, or more specifically of intrinsically disordered proteins (IDPs) or intrinsically disordered regions (IDRs) upon contact with interaction partners (137, 144, 145). Assuming millisecond HDX-MS dynamics (146), the increased application of MD simulations for HDX-MS data on multicomponent complexes is a veritable avenue. Other challenges lie in increased examples on HDX-MS on structural and dynamic characterization of integral membrane proteins and their interactions with ligands (147, 148) and moving from a few single proteins in solution to more complex assemblies *in vivo* as highlighted in recent studies (99, 100). As for XL-MS, we see that the field will be migrating from DDA-based HDX-MS to DIA-based HDX-MS for better quantitation (111), combined with an increased software development for automated data analysis and higher throughput (111, 131). On the level of integrated structural biology, we envision increased examples of combining HDX-MS with NMR spectroscopy to study dynamic and fuzzy complexes (Textbox 2) and the increased integration of HDX-MS with modeling and docking tools and especially also for dynamic regions and complexes (Textbox 2).

### Limited Proteolysis Mass Spectrometry (LiP-MS)

Much as HDX-MS, limited proteolysis mass spectrometry is a powerful method to probe for conformational changes in proteins and the dynamics of protein interactions (with ligands) (6). As for XL-MS and HDX-MS, information on dynamic and conformational changes in complexes revealed by altered proteolytic marks is invaluable for the modeling of proteins and the modeling and docking of protein complexes. These dynamic and conformational changes might arise from the interactions of protein-ligand complexes or by varying the experimental conditions, or alternatively, upon two or more proteins binding to each other in a complex, rendering specific regions inaccessible for proteolysis. While LiP-MS for protein-ligand interactions is more established, LiP-MS for protein-protein interactions is a more recent and challenging development (149).

**Fig. 4:**
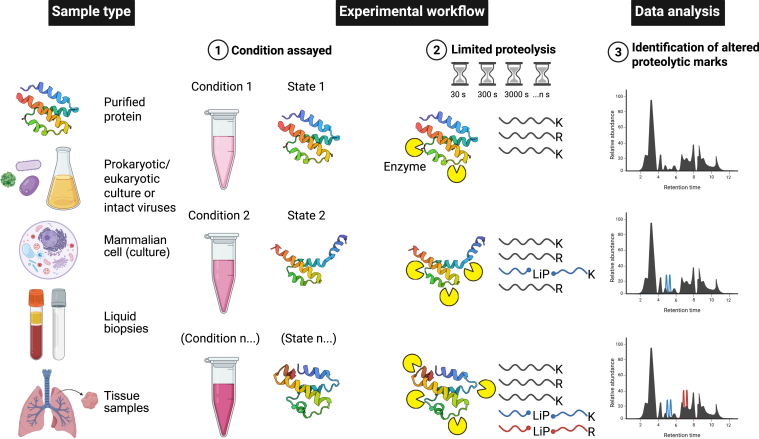
**Overview of the sample requirements, experimental workflow, and data analysis for limited proteolysis mass spectrometry.** LiP-MS can be applied for a broad variety of sample types, including purified proteins or protein complexes in solution, prokaryotic and lower eukaryotic cultures, intact viruses, mammalian cells (in culture), diverse liquid biopsies, and isolated tissue samples. In the experimental setup, the sample to be analyzed is incubated in a suitable buffer at a suitable temperature, and proteolytically digested to peptides at a defined set of time points. The peptides are identified and quantified by liquid-chromatography tandem mass spectrometry (LC-MS/MS; MS^2^), followed by data analysis using dedicated software and data interpretation. The typical data generated in an LiP-MS experiment yields signature peptides or altered proteolytic marks. Protein A: generic protein Z (Biorender icon), protein B: complement system C5a-desArg; state 1 - PDB ID: 4P3B (323), state 2 - PDB ID: 3HQA (324), and state 3 - PDB ID: 1KJS (325). Created in https://BioRender.com.

As for XL-MS, LiP-MS can be applied across the entire molecular scale from individual proteins in solution to larger protein machinery and entire cells (Fig. 3). Indeed, LiP-MS has, since its advent to map PPIs approx. 10 years ago (149), been applied to various biological systems, including microbes, mammalian cell lines, and tissues, as well as body fluids, as recently reviewed by Leene *et al.* (6). LiP-MS employs a controlled proteolytic digestion of proteins under near-native conditions to probe their local and global conformational dynamics (150). A broad-specificity protease (proteinase K, elastase, pepsin etc.) is used to preferentially cleave solvent-exposed and flexible regions, reflecting the protein’s conformation by distinguishing disordered or unfolded regions from folded regions or domains (Fig. 4). As a protein undergoes conformational shifts, binds a ligand, or forms a larger protein assembly, the exposed and protected regions (might) change, creating a distinct and measurable pattern in the protease cleavage pattern. LiP-MS relies on measuring changes in peptide patterns using standard peptide-based bottom-up proteomics (18) with no requirements for dedicated software outside of the scope of peptide identification. By comparing these patterns, the altered proteolytic marks, LiP-MS provides indirect structural and dynamic information about the protein’s state (Fig. 4). As this method is straightforward and simply relies on incubating the sample(s) with a given (set of) protease(s) for an increasing period of time, it is amenable to analyzing challenging protein systems in their native forms and can reveal critical structural transitions underlying biological regulation. Integrating LiP-MS findings with complementary structural data, such as cryo-EM models or predicted protein models (124, 151, 152), further refines our understanding of protein architectures and supports hypothesis-driven studies of protein function and regulation.

### Current State-of-the-Art in LiP-MS

During the past decade, LiP-MS has been used to characterize integral membrane proteins (capturing conformational changes across both flexible loops and transmembrane domains) (153) and to detect protein–protein interactions while pinpointing their interaction interfaces (150). This makes LiP-MS suitable for studying native multiprotein complexes and assemblies (potentially including protein–nucleic acid complexes) or low-abundance species. An emerging strength of LiP-MS is its potential for large-scale, proteome-wide analyses (154). Quantitative LiP-MS experiments can map conformational changes across an entire proteome, pinpointing regions that are sensitive to various stimuli, such as environmental changes, *e.g*., structural alterations during cellular stress or aging (155), drug binding, ligand-induced conformational shifts that reveal drug target sites (156), disease-related variants, and mutations or modifications associated with disease that cause detectable structural differences (6, 150, 157, 158, 159). This global view enables the discovery of key structural motifs or conformational “hotspots” that govern protein function, stability, or interaction specificity. Finally, the development of the LiP-Quant platform (156, 160), which integrates LiP-MS with machine learning, has enhanced the ability to identify drug targets and their binding sites in complex proteomes, hence advancing the field of chemoproteomics.

### Future Developments in LiP-MS

In its simplicity, LiP-MS is broadly applicable across the entire molecular scale from individual proteins in solution to larger protein machineries and entire cells. We see that the challenges for LiP-MS over the next 5 years reside in generating a similar common set of field recommendations and standardization as exists for XL-MS (34) and HDX-MS (5). Whereas there are several examples for integrating XL-MS and HDX-MS data with complementary proteomics and structural biology methods, as highlighted in Textboxes 1 and 2, these are scarcer for LiP-MS (Textbox 3). Here, we see several possibilities in providing an increasing number of examples, where LiP-MS has been used together with complementary methods. Moreover, future development for LiP-MS lies in the increased integration of LiP-MS with modeling and docking tools and especially for dynamic regions and complexes; the increased application of MD simulations for LiP-MS data on multicomponent complexes.

### Other Peptide-Level Structural Proteomics Methods

#### Mass Spectrometry-Based Protein Footprinting

Mass spectrometry-based protein footprinting uses solution-phase labeling strategies to probe a protein’s higher-order structure. In these experiments, reagents (or solvent isotopes) modify amino acid side chains or backbone amides that are exposed to solvent, effectively “marking” the protein’s solvent-accessible surface area (SASA) (8). One such example is deuterium labeling of backbone amides in HDX-MS experiments discussed above. Other, more rarely used, footprinting approaches use covalent chemical labels (such as hydroxyl radicals or residue-specific modifiers) that irreversibly target accessible amino acid side chains or that generate identifiable footprints by reactions with fast, irreversible labeling species that are highly reactive and hence footprint broadly several amino acid residue side chains on the time scale of submilliseconds (8, 161, 162). Notably, while HDX-MS is centered around reversible deuterium labeling of backbone amides and hence prone to back-exchange issues, hydroxyl radicals targeting protein side chains are irreversible, overcoming such problems. The identification of the labeled sites with bottom-up MS allows for the mapping of protein folding and monitoring conformational changes, or even the comparison of free vs. complexed proteins to pinpoint interaction interfaces based on changes in labeling. MS-based protein footprinting hence provides a relatively high-resolution view of protein structure and dynamics in solution, complementing classical structural biology techniques.

#### Fast Photochemical Oxidation of Proteins (FPOP)

FPOP is a hydroxyl radical protein footprinting technique in which a short laser pulse photolyzes hydrogen peroxide to generate hydroxyl radicals (•OH) that covalently oxidize solvent-exposed amino acid side chain (163). The resulting oxidative modifications are identified by MS, providing a readout of side-chain solvent accessibility at the residue level. As labeling occurs on the microsecond timescale, FPOP can capture transient conformations and fast folding events that might elude slower methods; it thereby offers complementary structural insights alongside techniques like HDX-MS (164, 165).

FPOP has been applied to systems ranging from small proteins to large complexes and even intact cells in *in vivo* and *in situ* studies, where hydroxyl-radical labeling is performed in living biological contexts (166, 167). It has proven useful for probing protein folding and dynamics, mapping membrane protein topology, and identifying protein–protein interfaces (*e.g.* antibody epitope mapping) (168, 169). Ongoing improvements in data analysis and workflows aim to enhance residue-level resolution and throughput, paving the way for proteome-wide FPOP experiments and further integration of this technique into the structural proteomics toolbox (163).

#### Covalent Protein Painting (CPP)

Protein painting was developed approximately 10 years ago, around the same time as LiP-MS for PPIs, and is designed specifically to reveal protein–protein or protein–drug interaction surfaces that are often hidden in complexes (13). It uses small organic dye molecules as molecular paints to coat the solvent-exposed surfaces of proteins in a preformed complex. The paint essentially blocks potential trypsin cleavage sites, excluded by the binding/interaction interface. Following standard bottom-up MS, only peptides hidden in interfaces or binding pockets emerge as positive hits, hence revealing the functional contact regions (13). By revealing these solvent-excluded interface peptides, protein painting provides a powerful approach to study the architecture of protein complexes and how subunits interact, and has to date been used to study *e.g.* interactions between nicotinic acetylcholine receptors (nAChRs; ligand-gated ion channel receptors contributing to *e.g.* to cognition, memory, and motor control) and their ligands (170), signaling in the PD-1/PD-L1 checkpoint pathway (171, 172) central in regulating immune responses, and osteoarthritis pathology (173) and in Alzheimer’s disease (174).

## Protein-Level Structural Proteomics Methods

### Top-Down Proteomics

Unlike bottom-up MS, top-down proteomics is focused on analyzing intact proteins. Top-down proteomics preserves the integrity of the protein(s) during analysis, hence maintaining critical information about PTMs and proteoform diversity, which is often lost in bottom-up MS approaches (175). This enables more comprehensive characterization of proteoforms and protein modification patterns, complementing bottom-up methods by providing deeper insight into how PTMs and sequence variations coexist on intact protein molecules (176, 177, 178). The advantages of top-down proteomics for proteoform and PTM identification include, *e.g.*, the preservation of the PTM context by avoiding the peptide-to-protein inference problem distinguished by bottom-up MS, by retaining the structural context of modifications on a protein (322). Top-down proteomics is particularly useful for the identification of intact proteoforms, where sequence variants, splice isoforms, or truncated proteoforms that share many peptides (and might be indistinguishable in bottom-up analyses) can be individually characterized, and for the detection of coexisting proteoforms for the same protein. As for XL-MS and HDX-MS, recommendations and best practices for intact protein analysis for top-down MS exist (179).

### Native MS

Much as top-down MS, native MS involves measuring intact proteins and protein complexes (180, 181, 182); however, in contrast to top-down MS, native MS is performed under minimally denaturing conditions, usually using electrospray in volatile, aqueous ammonium acetate buffers (183). Since its foundation, native MS has enabled the direct detection of quaternary structures and noncovalent interactions, offering an unparalleled view of oligomeric state, ligand binding, and proteoform microheterogeneity (184). A key aspect of native MS is that many proteins can retain their near-physiological folds and assemblies in the gas phase (185). Early applications centered on smaller complexes and stable viral capsids (186, 187); however, improvements during the past decade in instrumentation, especially high-resolution mass analyzers, have pushed the upper mass limits to the MDa range (184, 188) and permitting explicit quantitation of therapeutic monoclonal antibodies (mAb) (189), bound cofactors (190, 191), stoichiometries (192), and PTM variants (193).

A critical value of native MS lies in its ability to distinguish and quantify co-existing species that differ by very small, but non-zero, mass increments (*e.g.*, single hexose or fucose additions) and to baseline-separate near-isobaric proteoforms in a single spectrum. True isobars (identical mass) cannot be differentiated by intact-mass measurement alone; in such cases, native MS is typically coupled with gas-phase dissociation (*e.g.*, surface-induced dissociation (SID) or ECD) or to ion-mobility/ultra-high-resolution workflows to assign the modification. Using high-resolution FT-ICR or Orbitrap instrumentation, mass differences as small as 1.0 Da on a 150 kDa IgG have been resolved (194), enabling direct quantitation of >100 co-existing glycoforms or drug-to-antibody ratio (DAR) species. Applications for native MS indeed range from studies on antibody-drug conjugates (ADCs) and glycoprotein biosimilars, to multi-subunit machines such as ribosomes (195) and 20S proteasomes (196). Native MS complements orthogonal methods such as cryo-EM, NMR spectroscopy, X-ray crystallography, and small-angle scattering methods, providing snapshots of an assembly’s heterogeneity and binding equilibria under near-native conditions.

## Experimental Approaches in Interaction Proteomics

### Affinity-Purification MS (AP-MS)

AP-MS is one of the most widely used approaches for identifying PPIs on a large scale and remains a cornerstone of interaction proteomics (11). In this technique, a protein of interest (POI, the bait) is tagged with an affinity handle (*e.g.*, FLAG, Strep-tag, His, or novel tags such as ALFA) (197) and expressed in cells under mild lysis conditions. The tagged POI and its bound partners (preys) are isolated using an affinity matrix, washed to remove non-specific proteins, then enzymatically digested for bottom-up MS identification. As AP-MS relies on relatively stable interactions, it can miss transient complexes or membrane proteins requiring harsher solubilization. Nevertheless, recent advances in tag design, including optogenetically controlled systems like PhyB/PIF6 (198), as well as the combination of AP with cross-linking or labeling, have enhanced specificity and enabled more gentle elution strategies (199). AP-MS continues to be a robust platform for systematically mapping interactomes (Fig. 1 and Textbox 1, Textbox 2, Textbox 3), as demonstrated by large-scale projects that characterize hundreds of baits in human cells (200), aided by improvements in MS instrumentation and analysis software.

### Co-immunoprecipitation (Co-IP) MS

Co-immunoprecipitation (co-IP) MS is conceptually similar to AP-MS but typically uses an antibody against the endogenous bait protein (or a known epitope on the POI) rather than an engineered affinity tag (11). After mild cell lysis, the antibody–antigen complexes (along with any bound interacting proteins) are captured using Protein A/G agarose or magnetic beads, washed, and processed by bottom-up MS analysis. This approach can preserve native expression levels of the POI and circumvent the need for tagged constructs. However, co-IP performance depends heavily on the availability of high-affinity, highly specific antibodies that do not disrupt relevant interfaces. Despite these challenges, co-IP MS remains an important method for validating interactions discovered in large-scale screens or for studying endogenously expressed proteins under near-physiological conditions (201, 202) (Fig. 1 and Textbox 1, Textbox 2, Textbox 3).

### Limitations to AP-MS and Co-IP

Co-immunoprecipitation (co-IP) enriches endogenous complexes by capturing an antigen–antibody complex on Protein A/G resin, washing away nonspecific proteins, and analyzing the eluted material by bottom-up MS. As the bait is expressed at its native level, co-IP avoids over-expression artefacts, but its success hinges on high-affinity antibodies that do not disrupt the interaction interface (11). Multiple washing and elution steps are needed to reduce background proteins; however, these steps can cause labile or weak complexes to dissociate, meaning that pulldown-based technologies (AP-MS, co-IP, tandem affinity purification (TAP), etc.) are incompatible with determining binding affinities (11). Tags used for affinity purification may also mislocalize or destabilize the bait proteins in the target cells, and high-abundance contaminants often co-purify (11).

### Native Holdup (nHU)

The native holdup assay was devised to measure equilibrium binding affinities of full-length proteins directly from cell extracts. In nHU, biotinylated baits are immobilized on streptavidin resin at high concentration and incubated with a dilute cell extract (203). As the bait is in large excess relative to the prey proteins, the cumulative amount of bound prey consumes only a negligible fraction of the bait (203). After an equilibrium is reached, the liquid phase is separated rapidly by filtration or brief centrifugation, and the unbound fraction is quantified with MS. Apparent dissociation constants are then calculated by comparing prey depletion in bait versus control samples using a hyperbolic binding equation (203). Typical bait concentrations of ∼5 to 20 μM ensure accurate affinity estimates across a wide range (204, 205). Unlike traditional pulldowns, nHU measures prey depletion rather than bound complexes and eliminates washing steps, preserving weak interactions (206, 207). The assay can provide affinity estimates for thousands of endogenous full-length proteins in one experiment (205) and shows good agreement with fragmentomic measurements (203).

### Proximity Labeling (PL) MS

Proximity Labeling (PL) approaches address some limitations of AP-MS by capturing PPIs *in vivo* through covalent tagging of nearby proteins (typically within 1–20 nm) (208, 209, 210). A promiscuous enzyme (*e.g.*, BioID, APEX) is fused to the bait protein, and upon addition of a substrate such as biotin, a reactive intermediate is formed that labels proximal proteins with biotin (211). These labeled proteins are then purified on streptavidin beads and identified by bottom-up MS. Because no stable binding in cell lysates is required, PL excels at capturing dynamic or transient interactions, membrane-associated proteins, and subcellularly restricted assemblies (Fig. 1 and Textbox 1, Textbox 2, Textbox 3). Different enzymes offer varied labeling kinetics and specificities, from classic BioID (requiring ∼16–18 h) to evolved variants like TurboID, miniTurbo, AirID, or UltraID, which can label within minutes (209, 212, 213). Notably, PL can introduce background noise from bystander labeling and requires careful controls (*e.g.*, catalytically dead mutants, subcellular reference points) (211). Nevertheless, ongoing refinements, such as split-enzyme methods (214) or optogenetic variants (215) are expanding PL-MS utility for detecting even weak or short-lived protein interactions.

Beyond enzyme-driven PL, several platforms now harness photosensitizers that generate short-lived singlet oxygen (^1^O_2_) upon visible-light irradiation to covalently tag proteins within ∼10 to 20 nm radius. Genetically encoded miniSOG fusions (RinID/PDPL) or LOV-domain variants produce ^1^O_2_ *in vivo*, enabling millisecond-resolved mapping of dynamic interaction networks without exogenous H_2_O_2_ or long labeling incubations (216, 217). Small-molecule or antibody-tethered photocatalysts have further been adapted in the μMap platform, extending light-gated labeling to cell-surface and organelle microenvironments with high spatial precision (218). Singlet-oxygen approaches minimize background, allow reversible on/off control via illumination, and complement biotin-ligation enzymes by capturing transient or diffusion-restricted encounters, making them a valuable addition to the PL-MS toolkit.

### Integration of AP-MS and PL-MS

Although AP-MS enriches stable complexes and PL-MS excels at identifying transient or spatially restricted PPIs, these approaches often yield complementary subsets of interactors (11). The divergence arises from their underlying biochemistry: AP-MS involves cell lysis, affinity capture, and washing steps, so it favors stable, high-affinity interactions and can lose transient or detergent-sensitive partners. By contrast, proximity-labelling techniques use enzymes or photosensitizers fused to the bait to covalently tag proteins within a ∼10 to 20 nm radius in living cells, capturing transient, weak, or co-localized neighbors that would not survive purification (11). Because one method enriches intact complexes while the other tags the local microenvironment, they typically recover distinct subsets of interactors. Hence, combining AP-MS and PL-MS provides a more holistic overview of a protein’s interaction landscape. An emerging trend is to combine AP and PL workflows, either by generating separate constructs for each method or through single constructs (*e.g.*, the MAC-tag, which fuses Strep-tag for AP to BirA∗ for PL) (219, 220, 221). Such dual strategies can give a more holistic overview of POI’s interaction landscape. Integrative AP and PL approaches, aided by state-of-the-art MS and bioinformatics, now enable comprehensive mapping of protein networks, revealing both stable core complexes and transient associations in the native cellular environment.

### Proteome-Wide Methods in Interaction Proteomics

Global or unbiased approaches such as co-fractionation MS (CF-MS) or thermal proximity coaggregation (TPCA)/thermal proteome profiling (TPP) probe the entire interactome without requiring a tagged bait (12, 222, 223, 224, 225). CF-MS typically separates complexes by size exclusion or other biochemical properties, and proteins that co-elute across multiple fractions are inferred to interact. By contrast, TPCA/TPP exploits the stabilizing effect that protein partners have on each other’s thermal denaturation curves (222). Proteins with correlated melting profiles are predicted to reside in the same complex. Both strategies enable proteome-wide mapping of endogenous networks under near-physiological conditions and can be adapted to time-course or multi-condition studies (226). Despite challenges in fractionation throughput and data deconvolution, these methods have significantly advanced our ability to interrogate large-scale structural changes and dynamic interactions *in vivo*.

## Complementing Methods in Integrated Structural Biology

### Single-Particle Cryogenic Electron Microscopy (Cryo-EM) and Cryo-Electron Tomography (Cryo-ET)

The advent of single-particle cryo-EM as a routine technique for near-atomic resolution structure determination and as a complement to X-ray crystallography and NMR spectroscopy has marked a major turning point in structural biology. Cryo-EM has proven invaluable for studying large protein complexes that exceed the practical size limits of NMR spectroscopy, and the crystallization demands of X-ray crystallography. In a standard single-particle cryo-EM experiment, a pure and monodisperse protein sample is applied to a cryo-EM grid, blotted to form a thin aqueous film, and vitrified by rapid plunging into liquid ethane. The process hence preserves the molecules in their near native states. The data is collected using a transmission electron microscope (TEM) equipped with a direct electron detector, capturing thousands to millions of two-dimensional projection images of the molecules studied, across thousands of micrographs. These images are motion-corrected, and a contrast transfer function (CTF) is estimated, followed by particle extraction, 2D classification, and 3D reconstruction using iterative alignment and refinement algorithms. Advanced image processing pipelines also allow for the separation of conformational states, improving structural resolution and enabling the analysis of dynamic or heterogeneous assemblies. MDa assemblies such as ribosomes (58, 227, 228), proteasomes (229, 230), spliceosomes (231, 232), and membrane-bound receptors (233, 234) have been resolved to (near-)atomic detail, unveiling intricate architectures and facilitating the discovery of novel regulatory mechanisms. Many of these complexes play central roles in fundamental cellular processes such as translation, protein degradation, and signal transduction, and their structural characterization has provided key insights into their functional cycles and substrate specificity (235). In contrast to X-ray crystallography, the ability of cryo-EM to tackle heterogeneous samples composed of multiple conformations or subpopulations, allows for the isolation of distinct states for subsequent structural characterization. Time-resolved variants of cryo-EM (236, 237, 238), allows for the capture of short-lived (reaction) intermediates, providing a more complete picture of mechanistic pathways. When combined with structural proteomic techniques, cryo-EM data can be further validated and refined (Textbox 1, Textbox 2, Textbox 3). These integrative workflows confirm and fill in missing domains, confirm and resolve ambiguous interfaces, help resolve and define loops and flexible domains not clearly resolved in the electron density map, and in general lend confidence to pseudo-atomic models derived from lower-resolution densities, with specific examples highlighted in Textbox 1, Textbox 2, Textbox 3.

Cryo-electron tomography (cryo-ET), on the other hand, is a powerful approach for visualizing macromolecular assemblies directly within their native cellular environments, bridging the gap between isolated complexes and their functional contexts. As cryo-ET offers the possibility to study proteins in a native context that is often lost *in vitro* and hence provides the opportunity for *in situ* structural biology (239, 240, 241); possibly complemented by other integrated methods. Advances in subtomogram averaging methods now achieve improved resolution for *in situ* complexes (239, 240, 242, 243, 244), making it possible to resolve subunit organization, binding interfaces, and conformational changes (245) directly within their physiological milieu.

### NMR Spectroscopy

NMR spectroscopy exploits the magnetic properties of atomic nuclei (commonly ^1^H, ^13^C, ^15^N) to obtain detailed information on protein structure, dynamics, and interactions in solution (246, 247). Proteins labeled with stable isotopes (*e.g*., ^13^C, ^15^N) are often studied using multidimensional NMR experiments that resolve individual resonances corresponding to specific backbone or side chain nuclei (248, 249). Chemical shifts, coupling constants, and nuclear Overhauser effects (NOEs) can be used to derive distance and angle constraints, enabling *de novo* determination of high-resolution structures. Relaxation measurements provide information on local mobility and conformational exchange, making NMR especially powerful for studying intrinsic disorder and dynamic protein regions. NMR typically requires concentrated protein samples (often tens of milligrams) and suffers from molecular weight limitations in comparison to cryoEM and X-ray crystallography, with the practical upper limits around 30 to 50 kDa for routine studies.

In addition to mapping protein 3D structure(s) of individual proteins, NMR spectroscopy can be used to mapped protein–ligand and protein–protein interactions. Chemical shift perturbations (CSPs) upon ligand titration or complex formation reveal binding interfaces and conformational changes. Paramagnetic relaxation enhancement (PRE) or pseudocontact shifts can pinpoint distant binding sites or detect weak/transient interactions (250). NMR data often integrate with structural proteomics workflows to refine docking models or confirm dynamic loops/IDRs. These integrative workflows build on the use of cross-link distance constraints or HDX protection levels to complement NMR restraints, further confirming and pinpointing dynamic, solvent-exposed regions (Textbox 1, Textbox 2, Textbox 3). In-cell NMR extends studies to near-physiological contexts, complementing *in vivo* XL-MS or *in situ* cryo-ET (251, 252, 253). A current limitation for in-cell NMR is the required high cell densities (∼1 × 10^8^ cells/ml) and isotopically labeled proteins to achieve sufficient signal intensity for analysis (254, 255, 256).

### X-ray Crystallography

X-ray crystallography remains one of the foundational techniques for high-resolution protein structure determination. Here, proteins (or protein complexes) are crystallized, and the diffraction pattern produced by X-rays passing through the crystal is measured (257). By analyzing the intensities and phases of the diffracted X-rays, an electron density map is computed, into which a protein’s atomic coordinates are modelled to derive a static 3D structure at atomic resolution. As for NMR spectroscopy, X-ray crystallography typically requires concentrated protein samples (often tens of milligrams) and suffers from molecular weight limitations in contrast to cryoEM. As cryoEM and NMR spectroscopy, X-ray crystallography provides atomic detail of proteins and protein complexes, pinpointing side-chain orientations, hydrogen-bonding networks, and metal-ligand interactions. This detailed information underpins mechanistic insights, particularly for enzymes, receptors, and drug-target complexes. Co-crystal structures with small molecules or peptides reveal binding modes and structure-activity relationships. This enables structure-based drug design and lead optimization (258, 259). When combined with structural proteomic techniques, data from X-ray crystallographic experiments can be further validated and refined. These integrative workflows build on the use of cross-link distance constraints to model flexible domains that might be unresolved in crystal structures (260), confirm and resolve ambiguous interfaces, help resolve and define loops and flexible domains not clearly defined in the density map (Textbox 1, Textbox 2, Textbox 3). Fragment or domain structures can be pieced together with cryo-EM (261, 262) or computational models to yield more complete assemblies.

### Small-Angle X-ray and Neutron Scattering (SAXS and SANS)

Small-angle scattering methods applying X-ray or neutron scattering (SAXS and SANS), provides the low-resolution models (10’s of Å in contrast to atomic resolution for X-ray crystallography, NMR and cryoEM), *i.e.*, particle dimensions, overall shape, and subunit organization of (a) biomolecule(s) in solution by measuring rays scattered at low angles (263, 264). The benefit of SANS in comparison to SAXS is that neutrons scatter from atomic nuclei, and importantly, hydrogen (^1^H) and deuterium (^2^H) have very different scattering lengths. This allows for the use of contrast variation experiments unique to SANS; *i.e.*, by partial deuteration of the sample or by adjusting the hydrogen/deuterium ratio in the solvent, specific components of a complex can be matched out or highlighted during an experiment, revealing positions of individual proteins in a given complex (265). In contrast to a crystallographic experiment, SAXS and SANS experiments are done in near-physiological solution conditions and accommodate a wide range of molecule sizes, without requiring protein crystallization (266). A major strength of solution scattering is its ability to probe protein flexibility and dynamics (267). As SAXS/SANS measure molecules in solution, they naturally capture any conformational heterogeneity or disorder present. Flexible linkers, unfolded regions, or multi-domain protein when sampled in different orientations will produce characteristic scattering signatures. SAXS has been extensively applied to IDPs and proteins with flexible domains, to quantify their expanded vs. compact conformations (268).

SAXS and SANS are often combined with high-resolution structural data and data from structural proteomics experiments in an integrative modeling framework. The use of cross-link distance constraints allows for the pinning of certain subunits together while SAXS/SANS data ensures that the entire complex matches the correct overall shape (269). Likewise, incorporation of HDX-MS data can aid in mapping flexible regions, which, together with SAXS/SANS, helps to build consistent structural models (270, 271). As part of integrative structural biology workflows, small-angle scattering is helping to paint a holistic picture of biomolecular structures—from static architectures to the ensembles of motions that underlie protein function.

## Challenges in Multimodal Data Integration

When combining diverse experimental modalities (*e.g.*, XL-MS, HDX-MS, LiP-MS, cryo-EM/ET, X-ray crystallography, NMR, and SAXS/SANS) and computational predictions (*e.g.,* AlphaFold/RosettaFold-based models, and other docking solutions) (Textbox 1, Textbox 2, Textbox 3), discrepancies in the generated models can occur (272). Such discrepancies might derive from uncertainty in the underlying data that might be sparse, noisy, ambiguous, or derived from a heterogeneous population (272). Integrating heterogeneous datasets, such as an 8 Å cryo-EM density map, XL-MS distance restraints at a defined false discovery rate (FDR), and domain models from AlphaFold filtered by predicted aligned error (PAE) thresholds requires a computational framework capable of representing each data type’s uncertainty and relative reliability within a unified scoring function (273). In integrative structural modeling, each dataset is translated into spatial restraints with probability distributions that reflect experimental precision and possible systematic errors (272). These restraints are jointly optimized to generate an ensemble of models consistent with the input data within their uncertainties (273, 274). Traditional methods to resolve uncertainty and discrepancies include *e.g.* iterative refinement using computational modeling, *i.e.*, adjusting the weights and priorities of different datasets can help identify a compromise model that better satisfies all constraints. In many integrative modeling platforms, each dataset (*e.g.* cross-link distance restraints, cryo-EM map features, small-angle scattering profiles) is assigned a weight reflecting data quality and expected resolution. These weights are then optimized under a scoring function to produce models that reconcile conflicting constraints as closely as possible (273, 275, 276). Furthermore, in ensemble modeling, *i.e.*, representing the structure(s) as an ensemble of conformations rather than a single static model can capture dynamic fluctuations and reconcile conflicting constraints that reflect different coexisting states (277, 278). Other methods to resolve uncertainty and discrepancies include focused follow-up experiments (back-to-the-lab-bench), *i.e.*, designing targeted experiments, such as mutagenesis of cross-linked residues, time-resolved cryo-EM, or additional NMR measurements, which can clarify ambiguous regions and support or refute specific structural hypotheses (71, 279, 280, 281, 282, 283). Recent strategies, such as AlphaFold’s integration of experimentally determined structures and MSA-derived co-evolutionary information, show that implicit weighting schemes can be highly effective (15, 284). This framework emphasizes that integration is not solely about resolving conflicting data but about quantitatively combining complementary evidence (272, 273). Moreover, while AlphaFold reliably self-assesses its accuracy, combining AlphaFold predictions with XL-MS data has been shown to be beneficial especially in flexible and in disordered regions (88, 274). Best practice further recommends withholding a subset of data, such as a fraction of cross-links or orthogonal biochemical data, as an independent validation set, ensuring that models generalize beyond the restraints used for refinement (285). Such computational integration, when paired with experimental co-application of techniques, maximizes the structural and mechanistic insight obtainable from modern structural proteomics.

Ultimately, the resolution of conflicting data fosters a deeper understanding of proteins as dynamic entities, with multiple interconverting conformations and context-dependent architectures. These conflicting data and discrepancies are likely to increase even at a higher rate than currently upon the field moving to *in situ* structural proteomics, and in the future, systematic frameworks for addressing challenges in multimodal data integration are needed.

## Computational Advances Enabling Structural Proteomics

The integration of computational methods with experimental techniques has ushered in a new era of structural proteomics. Advanced algorithms in ML, AI-, and MD-based workflows have transformed the way we approach protein structure determination, from sequence-based predictions to integrative modeling of complex assemblies. AI-based tools predict protein structures with remarkable accuracy, while an expanding suite of ML- and MD-tools support the refinement, validation, and interpretation of experimental data (14, 15, 16, 54, 87, 132, 133) (Textbox 1, Textbox 2, Textbox 3). Despite the fact that we have touched upon AI-based tools above in the respective sections for XL-MS, HDX-MS, and LiP-MS, we argue that computational advances enabling structural proteomics workflows, including protein structure prediction and modeling PPIs warrant a section on its own, as this is one area where we foresee main advancement and new applications within the next few years.

The most prominent development in protein structure prediction is the advent of deep learning-driven models, notably AlphaFold and RoseTTAFold (14, 15, 16, 132), which revolutionized the field by achieving near-experimental accuracy. Leveraging large-scale genomic data and evolutionary relationships, AlphaFold’s neural network architecture predicts the three-dimensional fold of a protein from its primary sequence (14, 15). This innovation effectively closed the gap between computational predictions and high-resolution experimental methods for many well-folded, monomeric proteins *in vitro.* Despite their impressive achievements, AI-based predictors have limitations. While AlphaFold excels at predicting well-structured proteins, certain classes of targets, such as IDPs and proteins with IDRs, dynamic loops, and flexible domain linkers, remain challenging (286, 287, 288). Moreover, the accuracy of predicted structures can decrease for protein regions lacking sufficient evolutionary information or for large macromolecular complexes with many subunits and complex arrangements. Beyond single-protein folds, the community has rapidly adapted AlphaFold, RoseTTAfold and related algorithms to predict protein-protein complexes, oligomeric states, and specific binding interfaces. Hybrid approaches that integrate evolutionary couplings, molecular dynamics simulations, and coevolutionary data further enhance the power of these tools (289, 290, 291, 292).

Molecular docking algorithms predict the most favorable binding pose of a ligand (small molecule, peptide, or protein domain) within a target binding site, given a receptor structure. Tools like AlphaFold3 (15, 293), AutoDock (294), HADDOCK (295), ClusPro (296), and Rosetta-based docking protocols (16, 132) are widely used for predicting protein–protein and protein–ligand complexes. These methods often start with a static model of the receptor and ligand, proposing candidate binding modes based on geometric, electrostatic, and hydrophobic complementarity, as well as knowledge-based scoring functions derived from experimentally solved complexes.

The integration of AI-based predictions with experimental techniques has rapidly evolved from a conceptual framework to standard practice. While experimental methods provide critical insights, computational approaches have become indispensable for interpreting and extending these data. As more data integration pipelines and validation frameworks are developed, we will likely see increasingly reliable hybrid models that combine the strengths of both experimental and computational approaches. In doing so, we open new avenues for studying complex assemblies, understanding dynamic transitions, and guiding rational design in pharmacological and biotechnological applications.

### Use of XL-MS and HDX-MS as Restraints in Molecular Dynamics Simulations

Integrating XL-MS and HDX-MS restraints with MD simulations extends structural proteomics from static snapshots to dynamic ensembles. Most amine-reactive cross-linkers impose Cα–Cα distance limits of ≤30 Å, with zero-length reagents giving even tighter constraints (297); longer spacers (>35 Å) exist but offer weaker structural discrimination due to larger distance constraint range.

Parkinson disease is associated with the aggregation of α-synuclein, which exists in multiple oligomeric states. The dimeric state of α-synuclein has been a subject of debate, and in a recent study (90) distance restraints from BS3 (<30 Å) and DMTMM (<16 Å) cross-links were incorporated into discrete MD simulations, yielding eight structural clusters and revealing a compact, β-sheet-rich dimer with potential pathogenic relevance. In another recent weighted-ensemble MD approach studying XylE, a bacterial xylose transporter and homologue to the human glucose transporters GLUTs 1 to 4, HDX-MS protection factors were used as collective variables to bias sampling toward conformers that reproduce experimental exchange patterns, markedly improving prediction of degrader-induced ternary complexes. The combination of HDX-MS and MD showed that protonation plus substrate binding switches the XylE transporter from outward-to inward-facing, whereas an inhibitor locks it in the outward state (298).

Despite these advances, careful parameterization is essential. Restraints derived from sparse cross-links are commonly implemented as flat-bottom dependent on side chain distances or on statistical potentials, and their force constants must reflect the cross-linker’s length and the flexibility of the protein (299), as discussed also in Textbox 1. Moreover, distances should be treated as restraints rather than strict constraints because cross-linkers sample a distribution of lengths and proteins are intrinsically flexible (300). Interpreting HDX data is similarly non-trivial: microsecond MD simulations of folded states often fail to reproduce exchange rates, and maximum-entropy reweighting or other post hoc corrections may be needed (301). Regardless of approach applied, there are fundamental timescale mismatches; HDX experiments typically probe solvent exchange on seconds to minutes (302) and cross-link formation requires interactions to persist long enough for covalent bond formation (300), whereas all-atom MD is usually limited to micro-to millisecond trajectories (303). Over-constraining simulations by enforcing every cross-link can trap the system in rare conformations (300); instead, ensemble modelling and advanced sampling methods such as weighted-ensemble MD or maximum-entropy reweighting (301) are being developed to integrate experimental restraints with longer-timescale dynamics. With these methodological approaches and their limitations in mind, the synergy of XL-MS/HDX-MS and MD could promise routine exploration of complex structures and their conformational energy landscapes.

## Structural Proteomics to Decipher Disease Mechanisms Reveal Therapeutic Targets and Guide Drug Discovery and Development

The insights gained from structural proteomics extend well beyond basic biology, influencing diverse aspects of translational science. As structural characterization of proteins and their complexes reveal the molecular underpinnings of disease, these discoveries can be leveraged to identify new drug targets (140, 262, 294, 304, 305, 306), guide inhibitor design, and inform therapeutic interventions (307, 308, 309, 310). Notably, emerging integrative approaches make it possible to connect molecular-level structure-function relationships with cellular and organismal phenotypes, paving the way for more precise and personalized biomedical solutions within precision medicine.

MS-based ligand-binding assays, such as HDX-MS, LiP-MS, TPP and CPP are increasingly used to screen potential inhibitors or stabilizers in a high-throughput manner (5, 34, 160, 179, 225, 304). Coupled with cryoEM, X-ray crystallography, NMR spectroscopy, or integrative modeling (Textboxs 1–3), these data refine predictions of ligand placement and inform subsequent rounds of structure-based optimization. When integrated with computational docking and MD simulations, these experiments help prioritize hits that not only bind tightly but also induce favorable conformational changes. Overall, the combination of MS-based screening with *in silico* approaches accelerate the drug development timeline, reducing costs and increasing the likelihood of clinical success. This synergistic approach bridges the gap between high-throughput biochemical screens and detailed structural insights, enabling early identification of promising lead compounds (Textbox 1, Textbox 2, Textbox 3).

Textbox 1 **Requirements of XL-MS and its integration with other methods**

**Experimental Considerations**

*Sample purity:* XL-MS can be applied to a range of different sample types from purified proteins to intact cells in culture (Fig. 2).

*Sample amounts:* For purified proteins: 1 to 10 μg per protein; for cells in culture: 100 μg of total protein. The required sample amounts depend largely on downstream sample processing (*e.g.*, enrichment of cross-linked peptides or chromatography-based fractionation), which might increase the required sample amounts.

*In vitro*/*in vivo*: Applicable to purified proteins *in vitro*, cells in culture under *in vivo*-like settings.

**Integration With Other Methods**

**HDX-MS**: Comparison of cross-linked residues with backbone protection profiles of purified proteins in solution to determine whether cross-links overlap with regions of high structural stability or dynamic conformational exchange.

*Considerations:* Larger amounts of protein(s) are needed for HDX-MS than XL-MS, works mostly *in vitro* using purified protein (complexes), although examples of *in vivo* HDX-MS are emerging. Two separate experiments and datasets are required, one for XL-MS and one for HDX-MS.

*Selected examples:* A description of the interaction of full-length immunoglobulin class G (IgGs) with the neonatal Fc receptor (FcRn) (326), interaction of a tyrosine phosphatase and an adaptor protein (327) and multimodal mass spectrometry-based strategy for in-depth characterization of antigen–antibody complexes (106).

**LiP-MS**: Use of LiP digestion patterns *in vitro* or *in vivo* to validate cross-linked sites, confirming whether the cross-link restricts protease accessibility or indicates solvent-exposed loop regions.

*Considerations:* The sample types and requirements for LiP-MS and XL-MS are largely overlapping; however, two separate experiments and datasets are required.

*Selected examples:* A structural characterization for calmodulin regulation of a cyclic nucleotide-gated channel (328), characterization of folding kinetics and misfolded states of the protein phosphoglycerate kinase (86), mapping of the contacts between a membrane-integral adenylyl cyclase and its modulators (329).

**PL-MS**: Correlate distance constraints with *in vivo* labeling data to distinguish true physical interactions from purely collocated proteins.

*Considerations:* Integration is feasible on the level of intact cells *in vivo* (in culture), and both PL-MS and XL-MS data acquired on complex systems require strict data filtering. PL-MS captures interactions within a range of 10 to 20 nm, whereas XL-MS works in the distance range of around 1 to 3 nm or less. Can be performed as one single or two separate experiments.

*Selected examples:* The development of a method termed PL/XL-MS, which can enrich for a subcellular compartment by PL and simultaneously identify interactions of multiple proteins from XL-MS data (74).

**AP-MS/co-IP**: Combine cross-link distance constraints with AP-MS or co-IP-derived interaction networks to refine multicomponent complex architectures.

*Considerations:* Feasible on the level of liquid biopsies or (mildly lysed) cells in culture, can be applied both separately from XL-MS, with data integration on the level of observed PPIs, or with AP or co-IP followed by XL-MS on the same sample.

*Selected examples:* Serial capture affinity purification (SCAP) combined with XL-MS contributing to distance constraints for integrative structural modeling (330), AP followed by XL-MS to map PPIs (331, 332).

**Native MS:** Correlate XL-MS data on multisubunit complexes with oligomeric states from native MS for an intersection of stoichiometric and interface information.

*Considerations:* Whereas XL-MS can be applied to a range of different sample types, native MS requires specific considerations (non-denaturing buffers, high sample purity, and concentration) to maintain the native structure and interactions. Hence, while these methods can be applied to study a distinct phenomenon, two separate experiments and datasets are required.

*Selected examples:* Characterization of structures and oligomerization behavior of the tumor suppressor protein p53 complexes (333), identification of intermediates and stable off-pathways of the SNARE complex assembly (334).

**TPP:** Correlate cross-links with shifts in protein thermal stability to highlight domains or interfaces whose stabilization or destabilization pinpoints functionally critical regions or potential allosteric sites.

*Considerations:* Feasible *in vivo* on cells in culture; however, two separate experiments and datasets are required.

*Selected example*: Identification of glucose-binding proteins involved in metabolic disease (335).

**CPP:** Use chemical proteomics profiling to map residue reactivities across complexes. Overlay these data with cross-linked interfaces to identify how complex formation or conformational changes alter chemical accessibility and reactivity patterns.

*Considerations:* Feasible *in vitro* on purified components or *in vivo* on cells in culture; however, two separate experiments and datasets are required.

*Selected examples:* The use of CPP to yield modeling restraints for mapping the location and orientation of subunits within protein assemblies (336), the use of diazirine reagents in CPP to obtain deep coverage of a mitotic kinesin (337).

**Chemical footprinting:** Compare cross-linked regions to footprinted sites that become protected or exposed upon complex formation. Matching cross-link-defined interfaces with decreased footprint reactivity confirms newly buried surfaces or inter-subunit contacts.

***Considerations:*** Feasible *in vitro* on purified components; two separate experiments and datasets are required. Alternatively, use dead end crosslinks (Fig. 2) as a footprinting readout, probing for exposed, but non-cross-linked peptides or smaller domains, omitting the need for separate chemical footprinting samples.

*Selected examples:* Application of hydroxyl radical protein footprinting (HRPF) and XL-MS to study the complement system control protein Factor H (338).

**CryoEM/X-ray crystallography:** Map cross-links onto 3D densities to confirm subunit positions and resolve ambiguous interfaces in medium-resolution cryo-EM or partial X-ray structures.

*Considerations:* Feasible on the level of purified protein complexes *in vitro*. The sample requirements for cryoEM and XL-MS are largely overlapping, whereas X-ray crystallography depends on larger quantities (mg to tens of mg) of pure protein. Consider using XL to stabilize complexes prior to single-particle cryoEM.

*Selected examples:* Structural determination of core members of the autophagy machinery (339), elucidating the molecular basis for the regulation of human phosphorylase kinase (340), and the molecular basis of mRNA delivery to the bacterial ribosome (341).

**NMR:** Use cross-links to complement distance restraints, particularly for flexible domains that might be challenging to analyze by NMR alone.

*Considerations:* Feasible on the level of purified protein complexes *in vitro*. As for X-ray crystallography, NMR requires larger quantities (mg to tens of mg) of pure, often labeled sample.

*Selected examples:* Determination of the interaction patterns between Tau and 14-3-3ζ in Alzheimer’s disease (342), membrane interactions of α-synuclein involved in Parkinson’s disease (343).

**SAXS/SANS**: Correlate XL-MS data on multisubunit complexes onto the global envelope, improving shape interpretations in solution.

*Considerations:* Feasible on the level of purified protein complexes *in vitro*. As for X-ray crystallography, small-angle scattering methods require larger quantities (mg to tens of mg) of pure and perdeuterated protein in the case of SANS.

*Selected examples:* Integrative modeling of guanylate binding protein dimers (80), monitoring the time course of SAXS signals from cross-linked protein samples to assess the perturbation of chemical cross-linking on protein structure (344).

**ML- and AI-based modeling**: Incorporate cross-links as spatial restraints to guide integrative modeling, evaluate predicted protein-protein interfaces.

*Considerations:* When modeling or docking complexes based on XL-MS data, consider the length of the cross-linker spacer arm;a too-long spacer arm adds to the number of possible models due to flexibility, whereas a too-short spacer arm might not capture interprotein interactions.

*Selected examples:* Development of an integrated workflow employing generalized-correlation-based dynamic network analysis on multiple MD trajectories (81), molecular mechanism of fibroblast growth factor 2 oligomerization (92), structural studies of an apolipoprotein based on computational modeling and cross-linking (79).

Textbox 2 **Requirements of HDX-MS and its integration with other methods**

**Experimental Considerations**

*Sample purity:* HDX-MS can be applied to individual proteins or complex assemblies, optionally in the presence of a ligand (Fig. 3). A high sample purity is desirable but not essential.

*Sample amounts:* For standard experiments, 100 to 1000 μg of purified protein or complex is desirable.

*In vitro*/*in vivo*: Most applications to date are on *in vitro* systems, but *in vivo* HDX-MS examples are emerging.

**Integration With Other Methods**

**XL-MS**: Overlay amide-protection patterns from HDX-MS with cross-link distance constraints to confirm conformational regions that remain folded during complex assembly.

*For considerations and selected examples,* see Textbox 1.

**LiP-MS**: Compare HDX protection with protease-susceptible segments to verify which flexible domains are both rapidly exchanging and protease-accessible.

*Considerations:* The application of HDX-MS and LiP-MS is mostly feasible on *in vitro* systems; however, separate experiments and datasets are required. Larger amounts of protein(s) are needed for HDX-MS than for LiP-MS.

*Selected examples*: Characterization of conformational changes during protein phosphorylation (345), molecular mechanisms of conformational changes involved in the development of prion diseases (346), protein misfolding in von Willebrand disease (347).

**AP-MS/co-IP**: Map changes in HDX uptake onto interaction networks identified by AP-MS to link specific protected regions with known binding partners. Correlate co-immunoprecipitated complexes with HDX shifts to pinpoint interface regions stabilized upon immune capture or disrupted by mutations.

*Considerations:* When combining AP or co-IP experiments with HDX-MS, the former are most often used to identify the complexes to be studied in detail separately by HDX-MS. Hence, separate experiments and datasets are required for each method.

*Selected examples:* Development of histidine HDX to identify protein-drug interactions *in vivo* in cell lysates (348), identification of multiple host proteins modulating Ebola virus infection (349), integrative structural mass spectrometry demonstrating a host-pathogen PPI inducing local conformational shifts in plasminogen (350).

**PL-MS**: Relate HDX-protected segments to *in vivo* proximity-labeled sites, confirming whether newly tagged regions are buried or shielded in living cells.

*Considerations:* Whereas PL-MS works *in vivo* in cells in culture, HDX-MS is most often applied on the level of proteins or protein complexes *in vitro*. As mentioned earlier, when combining PL-MS experiments with HDX-MS, the former is suggested to be useful to identify the complexes to be studied in detail separately by HDX-MS.

*Selected example:* Characterization of the interaction of a tyrosine phosphatase and an adaptor protein (327).

**Native MS**: Combine HDX-derived flexibility data with oligomeric states from native MS to assess how subunit stoichiometry correlates with local structural protection.

*Considerations:* Both HDX-MS and native MS depend on relatively high sample purity and concentration to maintain the native structures and interactions of the protein(s) in complexes. However, separate experiments and datasets are required for each method.

*Selected examples:* Characterization of the binding mechanism of an epigenetic regulator for cancer therapy (351), development of an integrative molecular pharmacology and structural biology approach to study the mechanisms of GPCR hijacking by *Staphylococcus aureus* (352), and determining the dynamics of the assembly process for a viral capsid (353).

**TPP**: Integrate thermal unfolding transitions from TPP with HDX exchange rates to clarify whether global stability changes arise from localized flexible regions.

*Considerations:* While TPP is feasible *in vivo* on cells in culture, HDX-MS is typically still applied on isolated proteins and complexes *in vitro*. Two separate experiments and datasets are required on two different sample types.

*Selected example:* The development of protein thermal depletion (PTD) to reduce protein complexity prior to subzero-temperature HDX-MS for the high-throughput analysis of protein-ligand interactions in cell lysates (354).

**CPP**: Distinguish solvent-exposed loops identified by painting from HDX-protected elements, validating which surfaces remain shielded or become newly accessible.

*Considerations:* Combination of CPP and HDX-MS is feasible *in vitro* on purified components. These can be performed sequentially on the same sample, or as two separate experiments and datasets.

*Selected examples:* Validation that combining CPP with HDX-MS provides synergistic structural information on protein-ligand interactions (355), antibody-antigen complexes (356) and heat-stressed antibodies (357).

**Chemical footprinting**: Merge residue-specific chemical modifications with HDX exchange profiles to localize solvent-accessible side chains alongside backbone amide protection.

*Considerations:* HDX-MS is based on reversible labeling, whereas *e.g.* hydroxyl radicals used in footprinting are irreversible. Combine the sample in two different datasets to increase the resolution of the interaction interfaces based on changes in labeling.

*Selected references*: Dynamics of Aβ aggregation in Alzheimer's disease (358), structural changes of a thermally stressed monoclonal antibody (359), studies on prion protein misfolding (360).

**Cryo-EM/X-ray crystallography**: Use HDX data to resolve ambiguous densities by guiding domain orientations or flexible loops that are poorly defined in cryo-EM or crystallographic maps.

*Considerations:* The sample requirements for HDX-MS fall in between those for cryoEM and crystallization, whereas the sample purity requirements align.

*Selected references:* Determining the architecture, conformational dynamics, regulation, and specificity of ubiquitin ligases (151), characterizing structural dynamics of interactions within Dengue virus (152), and exploration of the conformational dynamics of the interaction of the enzyme glutamate decarboxylase (GAD) in complex with an autoantibody (124).

**NMR**: Integrate HDX protection levels with chemical shift perturbations or NOE patterns to confirm hydrogen-bonded motifs and dynamic exchange on fast timescales.

*Considerations:* As for X-ray crystallography, NMR requires larger quantities (mg to tens of mg) of pure, often labeled sample.

*Selected examples:* Discovery, analysis, and design of protein energy landscapes (100), construct optimization for protein NMR analysis (361), characterizing dynamic allosteric effects in the bacterial DNA sliding clamp due to ligand binding (362).

**SAXS/SANS**: Refine low-resolution scattering models by mapping HDX-identified flexible segments onto the global envelope, improving shape interpretations in solution.

*Considerations:* As for X-ray crystallography, small-angle scattering methods require larger quantities (mg to tens of mg) of pure and deuterated protein in the case of SANS.

*Selected examples:* Determining the structural basis for a receptor complex signaling (270), mapping the disorder in the Dengue capsid protein conformational ensemble (271) and the disorder-to-order transition of a *Bordetella pertussis* virulence factor upon binding calmodulin (363).

**ML- and AI-based modeling**: Map site-specific HDX protection onto predicted 3D models to validate backbone dynamics, refine ambiguous domains, or detect misfolded loops.

*Considerations:* Whereas HDX experiments typically probe dynamics over milliseconds to hours, conventional MD simulations often capture the nanosecond to microsecond timescale.

*Selected examples:* Determining the binding mode and mechanism of action of an atypical chemokine receptor (364), elucidating mechanisms of how N-glycosylation characteristics impact SARS-CoV-2 spike protein, resulting in increased surface accessibility (365), and predicting the basis of targeted protein degradation by combining MD simulations and HDX-MS (366).

Textbox 3 **Requirements of LiP-MS and its integration with other methods**

**Experimental Considerations**

*Sample purity:* LiP-MS can be applied to a range of different sample types from purified proteins to intact cells in culture, optionally in the presence of a ligand (Fig. 3).

*Sample amounts:* For purified proteins: 1 to 10 μg per protein; for cells in culture: 100 μg or more of total protein.

*In vitro*/*in vivo*: Applicable to purified proteins *in vitro*, cells in culture in *in vivo*-like settings.

**Integration With Other Methods**

**XL-MS**: Overlay altered proteolytic marks/signature peptides on cross-link distances to validate or refine 3D organization in multiprotein complexes.

*For considerations and selected examples,* see Textbox 1.

**HDX-MS**: Comparison of LiP-sensitive regions with backbone protection profiles to confirm unfolding events or ligand-induced protection patterns.

*For considerations and selected examples,* see Textbox 2.

**AP-MS/co-IP**: Identify structural rearrangements that regulate protein–protein binding surfaces; correlate LiP-induced proteolysis patterns with changes in captured complexes.

*Considerations:* LiP-MS can be used separately from or sequentially on AP or co-IP-enriched complexes.

*Selected example:* Mechanistic insight into a cyclin-dependent kinase inhibitor using orthogonal proteomics workflows (367).

**PL-MS**: Link LiP-MS results to *in vivo* interaction maps; sites that become protease-accessible might coincide with newly formed or disrupted contacts revealed by proximity tagging

*Considerations:* Both PL-MS and LiP-MS can be applied *in vivo* using cells in culture and applied separately on two different samples or, in theory, using LiP on proximity-labeled samples.

**Native MS**: Correlate LiP-induced proteolysis patterns with subunit stoichiometry from native MS to link flexible regions with shifts in quaternary assembly.

*Considerations:* While LiP-MS can be applied to a range of different sample types, as well as protein-protein or protein-ligand interactions, native MS requires specific considerations to maintain the native structure and interactions, as highlighted in Textbox 1. Hence, while LiP-MS and native MS can be applied to study a distinct phenomenon, they are rarely applied to the same sample preparation.

Selected example: Monitoring of protein O-GlcNAcylation by native MS and LiP-MS (368).

**TPP:** Combine LiP-MS with TPP to connect proteolysis-sensitive sites to changes in protein stability across temperature gradients.

*Considerations:* While both LiP-MS and TPP are feasible *in vivo* on cells in culture, consider generating separate datasets for each method rather than performing them sequentially to keep the data analysis workflows streamlined.

*Selected example:* Protein folding stability profiling of colorectal cancer for biomarker discovery (369).

**CPP:** Overlay LiP-MS cleavage sites with paint-protected surfaces to pinpoint newly exposed or solvent-excluded interfaces in protein complexes.

*Considerations:* While both LiP-MS and CPP are feasible *in vitro* and *in vivo*, consider generating separate datasets for each method rather than performing them sequentially to keep the data analysis workflows streamlined.

**Chemical footprinting:** Merge LiP-MS data with residue-specific chemical labeling to localize structural rearrangements and unfolding events at the amino acid level.

*Considerations:* Same as above for the combination of TPP and CPP.

*Selected example:* Structural changes in Protein G in response to changes in pH (370).

**Cryo-EM/X-ray crystallography**: Use LiP-MS to highlight flexible loops or conformational changes not clearly resolved in electron density maps or diffraction data.

*Considerations:* Feasible on the level of purified protein complexes *in vitro*, sample requirements for cryoEM and LiP-MS are largely overlapping, whereas X-ray crystallography depends on larger quantities (mg to tens of mg) of pure protein.

*Selected example:* Integrative structural modeling of the yeast exocyst complex (371).

**NMR:** Combine LiP-MS cleavage patterns with chemical shift perturbations or relaxation experiments to pinpoint dynamic, solvent-exposed regions.

*Considerations:* As for X-ray crystallography, NMR requires larger quantities (mg to tens of mg) of pure and often labeled sample.

**SAXS/SANS**: Use LiP-sensitive regions to refine low-resolution scattering models, linking flexible domains to shape distributions in solution.

*Considerations:* As for X-ray crystallography, small-angle scattering methods require larger quantities (mg to tens of mg) of pure, deuterated protein in the case of SANS.

**ML- and AI-based modeling**: Map LiP-cleavage events onto predicted or simulated structures to validate local flexibility or guide integrative refinements.

*Considerations:* LiP-MS captures conformational states under near-native conditions, including rare or low-population states that might be difficult to observe in conventional MD. The altered proteolytic marks from LiP-MS can be used to validate or refine MD simulations, and in contrast, MD predictions of potential altered proteolytic sites can guide experimental design, such as selecting specific proteases or probing structural states under different conditions.

*Selected example:* Mapping polymer interactions in bioconjugates via time-resolved LiP-MS (372).

In addition to guiding drug discovery and development, methods in structural proteomics provide an unprecedented view of how proteins fold, assemble, and undergo disease-related changes. Combining MS-based workflows with genomic, transcriptomic, and metabolomic profiles allows for the detection of subtle conformational alterations, unusual proteoforms, or aberrant PTMs that correlate with individual risk, prognosis, or treatment response (309). In many cases, protein malfunction arises from misfolding, aggregation, or abnormal complex formation rather than from changes in abundance. These readouts can reflect whether a given protein adopts pathologically relevant shapes or interactions that drive disease. HDX-MS, LiP-MS, TPP and XL-MS can capture these events under near-native conditions (135, 136, 144, 158, 159, 174, 311, 312) revealing local unfolding or complex assembly associated with particular mutations or splicing variants.

Such conformational biomarkers emerge in *e.g*. neurodegenerative diseases. Neurodegenerative disorders like Alzheimer’s and Parkinson’s, as well as certain systemic amyloidoses, involve misfolded proteins and amyloid fibril formation (136). These structural insights highlight potential therapeutic targets, such as solvent-exposed hydrophobic patches or critical β-sheet interfaces that promote fibril elongation (313). Small molecules, antibodies, or peptides designed to disrupt these interfaces can prevent aggregation, destabilize fibrils, or promote clearance of misfolded proteins (311, 312). Structural proteomics approaches make it possible to identify these altered interfaces and understand how changes in stability, binding affinity, or cooperativity contribute to disease. Here, LiP-MS or limited chemical labeling helps identify early-stage protein misfolding before aggregates become histologically evident (158). These structural fingerprints, altered proteolytic marks or labels, can improve diagnosis, track disease progression, and suggest novel therapeutic sites (150). By comparing normal versus pathological states of the same protein in different individuals, investigators may pinpoint how subtle local disturbances unfold into damaging fibrils, giving impetus to strategies that stabilize native folds in early disease or block toxic oligomers (157). In parallel, structural proteomics accelerates drug discovery by revealing where ligands bind, how they modulate protein flexibility, and whether they engage patient-specific variants (314, 315). MS-based target deconvolution also clarifies whether a candidate drug hits its intended protein or inadvertently binds another, reducing late-stage failures and guiding the design of combination therapies that address the heterogeneous reality of complex diseases (306, 316).

In short, structural proteomics is turning static abundance data into actionable three-dimensional insights, revealing how disease mechanisms differ from patient to patient and how novel drugs can precisely target those aberrant shapes or interfaces (135, 157, 158, 159, 317). As new MS chemistries, labeling strategies, and software approaches continue to develop, the structural dimension of proteomics will become integral to personalized and precision medicine (308, 318), enabling the prevention and treatment of disease through the fine-grained control of protein structure at an individual level.

## Future Directions in Structural Proteomics: Toward a Holistic View of the Cellular Proteome

The field of structural proteomics is advancing at a remarkable pace, propelled by emerging technologies that push the boundaries of resolution, throughput, and complexity. The focus should not only be on improving existing methods but also on developing new approaches that enable real-time, *in vivo* observations of proteins and their complexes. These advances should offer an increasingly holistic understanding of the proteome; its spatial organization, dynamic interplay, and functional adaptation under changing conditions, integrating structural data with proteomic networks, organellar architecture, and cellular ultrastructure (319, 320). Such efforts should pave the way for integrated structural workflows that capture proteins in their native states and directly link structural changes to functional outcomes. Future efforts should also focus on connecting structural insights to other ‘omics’ disciplines, such as transcriptomics, metabolomics, and lipidomics, to unravel the intricate regulatory systems that govern protein function and interaction dynamics in living cells. Achieving this holistic view will enable us to characterize the proteome not simply as a collection of static structures, but as a dynamic, interconnected network that responds to developmental cues, environmental changes, and disease states (305). The ultimate goal is to push beyond the structural proteomics methods discussed here in some detail, and the complementary integrated methods in structural biology briefly overviewed, to include spatial proteomics (320, 321) and big-science endeavors, here mainly referring to π-HuB (78), and combine these with cryoET and high-resolution microscopy of live cells, to understand how cellular architecture shapes the proteome’s structural states and dynamic transitions. In this context, systems structural proteomics will be a key enabler, allowing researchers to assemble proteome-wide structural maps and decipher emergent properties of protein networks in both healthy and diseased cells. By integrating XL-MS, HDX-MS, LiP-MS, spatial proteomics, and AI-enhanced modeling, the community can begin to decode how protein function and dysfunction unfold at multiple levels molecular, organellar, and cellular driving discoveries that bridge fundamental biology and translational research.

## Conflict of Interest

The authors declare that they do not have any conflicts of interest with the content of this article.
